# Long Noncoding RNA MMP2-AS1 Contributes to Progression of Renal Cell Carcinoma by Modulating miR-34c-5p/MMP2 Axis

**DOI:** 10.1155/2022/7346460

**Published:** 2022-03-16

**Authors:** Bo Fan, Yunfeng Niu, Zongtao Ren, Shufei Wei, Yongliang Ma, Jianzhi Su, Aili Zhang

**Affiliations:** ^1^Department of Urology, The Fourth Hospital of Hebei Medical University, Jiankang Road 12, 050011 Shijiazhuang, China; ^2^Laboratory of Pathology, Hebei Cancer Institute, The Fourth Hospital of Hebei Medical University, Jiankang Road 12, 050011 Shijiazhuang, China

## Abstract

Renal cell carcinoma (RCC) serves as a prevalent malignancy of urinary system and presents severe mortality and increasing incidence. Long noncoding RNAs (lncRNAs) have demonstrated critical roles in RCC development. Here, we were interested in the function of MMP2-AS1 during RCC progression. We observed that MP2-AS1 localized in both nucleus and cytoplasm of RCC cells using fluorescent in situ hybridization (FISH). The cell viability, proliferation, invasion, and migration of RCC cells were reduced by the depletion of MMP2-AS1. The MMP2-AS1 depletion-inhibited viability, proliferation, migration, and invasion of RCC cells were rescued by the overexpression of MMP2 *in vitro*. Consistently, the tumor growth of RCC cells was repressed by the depletion of MMP2-AS1 in the nude mice, while the overexpression of MMP2 could reverse this effect *in vivo*. Mechanically, we predicted the potential interaction of miR-34c-5p with both MMP2-AS1 and MMP2. The treatment of miR-34c-5p mimic reduced the luciferase activity of MMP2-AS1 and MMP2 3′UTR. The depletion of MMP2-AS1 enhanced miR-34c-5p expression and the expression of MMP2 was inhibited by miR-34c-5p in RCC cells. The protein levels of MMP2 were downregulated by MMP2-AS1 knockdown, while the inhibitor of miR-34c-5p rescued the expression of MMP2 in the cells. The treatment of miR-34c-5p mimic attenuated the cell viability, proliferation, invasion, and migration of RCC cells, in which MMP2 overexpression restored the phenotypes. MMP2-AS1 depletion-attenuated viability, proliferation, migration, and invasion of RCC cells were reversed by miR-34c-5p inhibitor. We concluded that MMP2-AS1 contributed to progression of renal cell carcinoma by modulating miR-34c-5p/MMP2 axis.

## 1. Introduction

Renal cell carcinoma (RCC) is one of the most commonly occurred malignancies of urinary system, which accounts for over 3% of human malignancies [1]. Over the past two decades, the incidence of RCC stably increases annually [2]. RCC is a heterogeneous malignant tumor form, mainly contains four subtypes, including the clear cell RCC (ccRCC), chromophobe renal carcinoma (chRCC), renal oncocytoma (RO), and papillary renal cell carcinoma (pRCC), among which, the ccRCC accounts for 70 to 80 percent of RCC [3]. ccRCC is derived from the epithelial cells of the proximal renal tubule, and its treatment remains a challenge in clinics owing to developed chemotherapy and radiotherapy resistance [3]. Currently, the surgical resection is the most prevalent choice for patients with RCC and the only potential treatment that results in a complete cure [4]. Hence, the exploration of regulatory mechanisms and development of novel therapeutic manner are urgently needed.

Long noncoding RNAs (lncRNAs) are RNAs with length more than 200 nucleotides and do not encode proteins [5–8]. Accumulating evidences have unraveled the role of lncRNAs as molecular regulators during numerous pathological and physiological processes, including the tumorigenesis, organ development, as well as immunity [9–11]. lncRNAs regulate gene expression and protein functions mainly through regulating decoys, tethers, scaffolds, and guides [12–14]. Studies also suggested that lncRNAs play important role during modulation of RCC. For instance, lncURRCC modulates histone H3 acetylation of EGFL7 promoter, which consequently activates AKT signaling and promotes cell proliferation and metastasis [15]. Liu and colleagues suggested that LINC00973 functioned as competing endogenous RNA (ceRNA) to modulate cell surface abundance of Siglec-15 and participated in cancer immune suppression of ccRCC [16]. lncRNA MMP2-AS1 is a poorly investigated lncRNA and the function of MMP2-AS1 RCC remains unreported.

MicroRNAs (miRNAs) also belong to noncoding RNAs and are characterized by short sequence of less than 20 nucleotides [17]. It has been widely accepted that miRNAs play pivotal roles in the regulation of cancer development, as well as drug resistance, by directly targeting mRNAs [18–20]. miR-34c-5p is able to inhibit Notch1 and represses invasion and metastasis in cervical cancer [21]. SIRT6 targeted by miR-34c-5p contributes to proliferation of colon cancer by JAK2/STAT3 signaling [22]. miR-34c-5p suppressed leukemia development and facilitates the eradication of leukemia stem cells [23]. A recent work also identified miR-34c-5p as a promising predictive biomarker of ccRCC patients, upon treatment with VEGFR-TKIs and even HIF-2*α* inhibitor belzutifan [24]. Moreover, MMP2 plays crucial roles in the development of RCC. It has been reported that miR-429/CRKL axis modulates the malignant progression of RCC by SOS1/MEK/ERK/MMP2/MMP9 signaling [25]. G6PD promotes invasion of RCC cells by inducing MMP2 expression via ROS/MAPK signaling [26]. However, the correlation of MMP2 and miR-34c-5p with MMP2-AS1 in the development of RCC is still elusive.

In this work, we identified a lncRNA, namely, lncMMP2-AS1, to be highly abundant in tumor samples collected from patients with RCC, determined its role in promoting cell proliferation and metastasis in *in vitro* and *in vivo* models. Studies on mechanisms identified a lncMMP2-AS1/miR-34c-5p/MMP2 regulatory axis in modulating cell behaviors. Our findings provided a novel therapeutic target for RCC treatment.

## 2. Materials and Methods

### 2.1. Cell Lines and Culture

The immortalized proximal tubule epithelial cell line HK-2 and RCC cell lines ACHN, A498, CAKI-1, OSRC-2, and 786-O cells were obtained from American Type Culture Collection (ATCC, USA). All cells were maintained in DMEM (Gibco, USA) that contains 10% fetal bovine serum (FBS) (Gibco) in a 37°C humidified environment filled with 5% CO_2_.

### 2.2. Cell Transfection

The small interfering RNAs (siRNAs) target lncMMP2-AS1 (si-MMP2-AS1), the miR-34c-5p mimics and miR-34c-5p inhibitors, the MMP2 overexpressing vector (pCMV-MMP2), and the corresponding negative control (NC) were synthesized by Gene Pharma (China). Cell transfection was conducted by using Lipofectamine 2000 reagent (Invitrogen, USA) according to the manufacturer's protocol.

### 2.3. Quantitative Real Time PCR

Total RNAs were extracted from RCC cells by using ice-cold TRIzol reagent (Invitrogen, USA) in line with the manufacturer's instruction. A total of 1 *μ*g RNA was reverse transcript to cDNA by using High-Capacity cDNA reverse transcription kits (Thermo, USA). Subsequently, the cDNA was adopted as the template for quantification of gene levels by using SYBR Green/ROX qPCR Master Mix (Thermo, USA). GAPDH and U6 were used as internal control for normalization of RNA and noncoding RNA, respectively.

### 2.4. Fluorescent In Situ Hybridization (FISH)

The localization of RNA was determined by FISH assay by using Fluorescent In Situ Hybridization Kit (Invitrogen, USA) according to the manufacturer's protocols. The Cy3-labeled lncMMP2-AS1 probe was purchased from RiboBio (China) and incubated with RCC cells. The fluorescence was detected under confocal microscopy (Leica, Germany).

### 2.5. Cell Proliferation

Cell proliferation was assessed by colony formation and cell counting kit-8 (CCK-8) (Beyotime, China). For CCK-8 assay, RCC cells (5 × 10^3^ cells/well) were transfected with indicated oligonucleotides, respectively, and then placed into 96-well plates. At indicated time points (24, 48, 72, and 96 hours), the CCK-8 reagent (20 *μ*l) was added into each well and cultured for another 2 hours. To measure the absorbance values at 450 nm, a microplate spectrometer (Thermo Fisher Scientific) was used.

For colony formation assay, RCC cells (1 × 10^3^/well) were suspended in complete culture medium and seeded into 6-well plates. After incubation for 2 weeks, visible colonies were stained with Crystal Violet (Beyotime), captured by a dissection microscope (Nikon, Japan) and counted.

### 2.6. Cell Migration and Invasion

Wound healing assay was used to assess cell migration of RCC cells. In brief, the RCC cells were seeded in 6-well plates and incubated overnight to form monolayers. Next day, a scratched area was created by using a 200 *μ*l pipette tip. After that, cells were washed with PBS to remove cell debris and cultured in media with 0.1% FBS. The spread of wound closure was photographed at 0, 6, and 12 hours.

Transwell assay was also performed to assess cell migration and invasion. In brief, cells were suspended in serum-free culture medium and placed onto the top chambers of 24-well Transwell plates coated with or without Matrigel for cell migration and invasion, respectively. And the lower chambers were filled with complete medium. After incubation for 24 hours, the invaded and migrated cells were fixed with 4% paraformaldehyde and then dyed with 0.2% crystal violet. Images were taken under a microscope.

### 2.7. Western Blotting

The total proteins were obtained by using RIPA buffer (Thermo, USA) that added with antiprotease mixture (Thermo, USA). Equal amounts of 35 *μ*g proteins were separated in SDS-PAGE gel, blotted onto PVDF membranes, blocked with 5% skim milk, and then hatched with specific primary antibodies against MMP2 (1 : 1000, Abcam, USA), cyclin D1 (1 : 1000, Abcam, USA), cyclin E (1 : 1000, Abcam, USA), E-cadherin (1 : 1000, Abcam, USA), Vimentin (1 : 1000, Abcam, USA), and GAPDH (1 : 1000, Abcam, USA) overnight at 4°C. The blots were then incubated with HRP-conjugated anti-rabbit secondary antibody at room temperature for 1 hour and visualized by using an ECL chemiluminescence substrate (Beyotime, China) in a gel image system.

### 2.8. In Vivo Xenograft Model

All animal experiments in this work were performed in line with the guidelines of Animal Ethic Committee of The Fourth Hospital of Hebei Medical University. Male SCID/nude mice (6-week-old) were brought from the Charles River Laboratory (USA). The transfected OSRC2 cells (5 × 10^5^) were suspended in 100 *μ*l Matrigel and subcutaneously inoculated in the fat pad of mice. Tumor volume was monitored every three days and calculated by the following equation: length × width^2^/2. All mice succumbed to death, and the isolated tumors were weighted and stored for following evaluation.

### 2.9. Histological Analysis

The collected tumors were made into 4 *μ*m thick paraffin sections for histological analysis. The tissue damage was assessed by hematoxylin & eosin (HE) staining following the manufacturer's instruction. Cell proliferation was detected by staining of Ki-67 and PCNA. In short, the samples were dewaxed in gradient ethanol, heated for antigen retrieval, hatched with 3% hydrogen peroxide, blocked with 5% BSA solution, and incubated with primary antibodies at 4°C overnight. The 3,3-diaminobenzidine (DAB) (Beyotime) was adopted as substrates for immunoperoxidase staining. The nuclei were counterstained with hematoxylin.

### 2.10. Luciferase Reporter Gene Assay

The wild-type (WT) or mutant (MUT) lncMMP2-AS or MMP2 3′UTR sequences was synthesized and cloned into pmirGLO vector, respectively. RCC cells were seeded in 12-well plates and cotransfected with WT or MUT luciferase plasmids and miR-34c-5p mimics or NC mimics. The pRL-TK vectors were cotransfected as internal reference. After 24-hour incubation, a Dual-Luciferase Reporter Assay System (Promega, USA) was adopted to evaluate the luciferase activity.

### 2.11. RNA Immunoprecipitation (RIP) with AGO2

The RIP assay was performed by using a Magna RIP RNA-binding protein immunoprecipitation kit (Millipore, Germany) in accordance with manufacturer's description. RCC cells were lysed and sonicated, incubated with Dynabeads precoated with AGO2 antibody or IgG antibody in RIP immunoprecipitation buffer overnight at 4°C. The RNA was then eluted, purified, reverse transcribed to cDNA, and subjected to qRT-PCR.

### 2.12. RNA Pulldown

Biotin-labeled wild-type (wt) miR-34c-5p (Bio-wt miR-34c-5p) and mutated (mt) miR-34c-5p (Bio-mt miR-34c-5p) probe were synthesized by Gene Pharma (China). RCC cells were transfected with the probes for 48 hours and harvested and lysed. Cell lysates were mixed with M-280 streptavidin magnetic beads (Sigma, USA) for 6 hours at 4°C. The beads were then washed, eluted, and subjected to qRT-PCR for lnc-MMP2-AS1 quantification.

### 2.13. Statistics

All statistical analyses were conducted by using the GraphPad Prism (version 17). Data were shown as mean ± S.D. Student's *t*-test or one-way analyses of variance (ANOVA) was performed for comparison between groups if they followed a normal distribution; otherwise, the nonparametric Mann–Whitney test was adopted. Differences were considered statistically significant for values of *p* < 0.05.

## 3. Results

### 3.1. The Depletion of MMP2-AS1 Suppresses Proliferation of Renal Cell Carcinoma Cells

**Figure 1:**
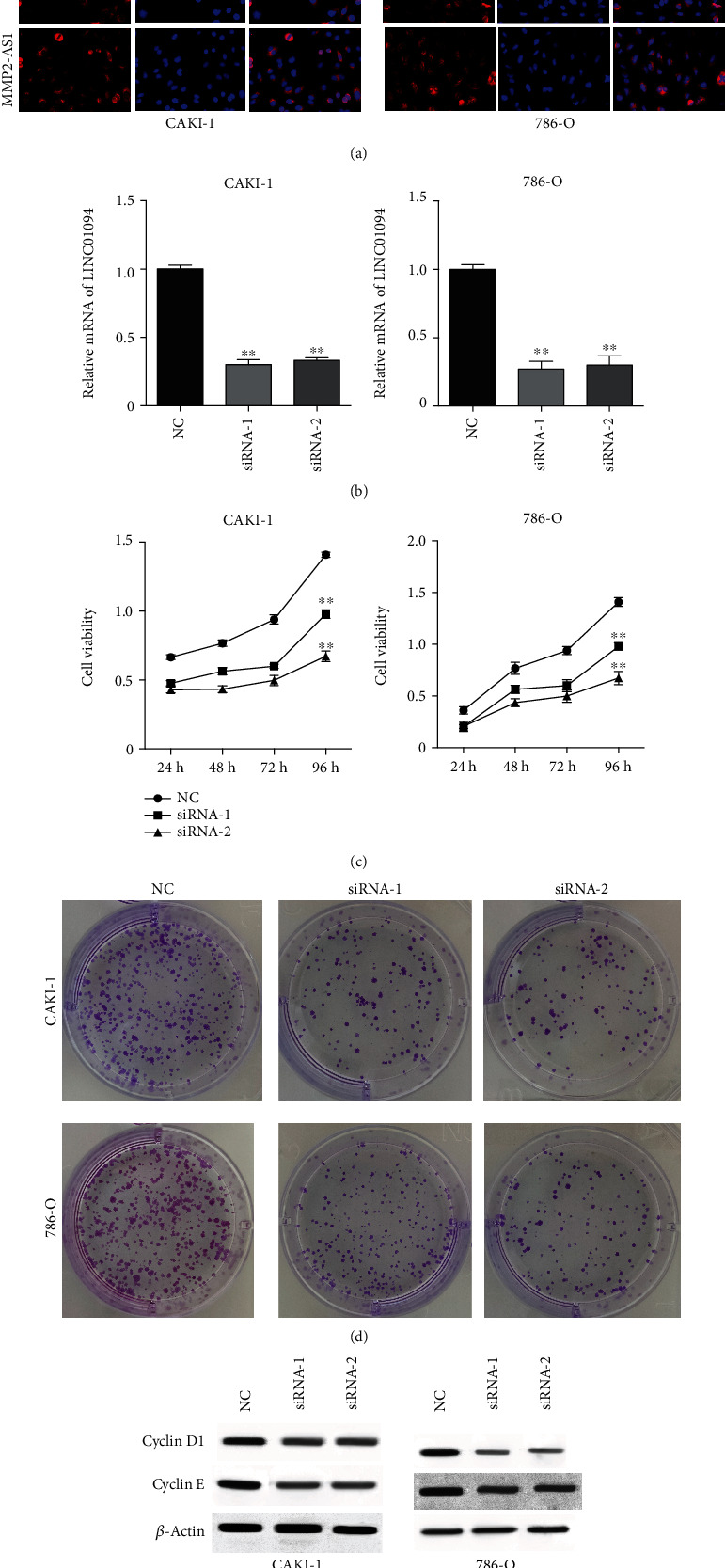
The depletion of MMP2-AS1 suppresses proliferation of renal cell carcinoma cells. (a) The localization of MMP2-AS1 was analyzed by FISH assay. (b–e) The CAKI-1 and 786-O cells were treated with MMP2-AS1 siRNAs. The expression of MMP2-AS1 was measured by qPCR. (c) The cell viability was detected by CCK-8 assay. (d) The cell proliferation was determined by colony formation assay. (e) The expression of cyclin D1 and cyclin E was detected by Western blot. ^∗∗^*P* < 0.01.

To assess the effect of lncRNA of MMP2-AS1, the CAKI-1 and 786-O cells were transfected with MMP2-AS1 siRNAs. MMP2-AS1 localized in both nucleus and cytoplasm of CAKI-1 and 786-O cells (Figure 1(a)). The depletion of MMP2-AS1 by siRNAs was validated in the cells (Figure 1(b)). The cell viability of CAKI-1 and 786-O cells was remarkably reduced by the depletion of MMP2-AS1 (Figure 1(c)). The knockdown of MMP2-AS1 significantly repressed the colony formation numbers of CAKI-1 and 786-O cells (Figure 1(d)). The expression of cyclin D1 and cyclin E was repressed by the depletion of MMP2-AS1 (Figure 1(e)).

### 3.2. The Depletion of MMP2-AS1 Represses Migration and Invasion of Renal Cell Carcinoma Cells

**Figure 2:**
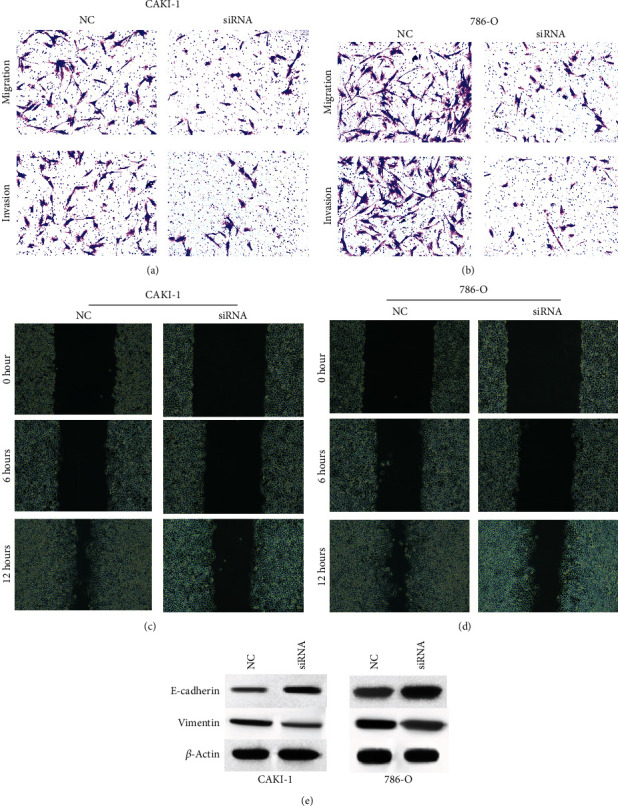
The depletion of MMP2-AS1 represses migration and invasion of renal cell carcinoma cells. (a–e) The CAKI-1 and 786-O cells were treated with MMP2-AS1 siRNA. (a, b) Cell migration and invasion were analyzed by Transwell assay. (c, d) Cell migration was detected by wound healing assay. (e) The expression of E-cadherin and vimentin was detected by Western blot.

We further analyzed the function of MMP2-AS1 in the modulation of migration and invasion of CAKI-1 and 786-O cells. We observed that the depletion of MMP2-AS1 remarkably attenuated the invasion and migration ability of CAKI-1 and 786-O cells (Figures 2(a) and 2(b)). Meanwhile, the knockdown of MMP2-AS1 significantly reduced wound closure rate of CAKI-1 and 786-O cells in the wound healing assay (Figures 2(c) and 2(d)). The E-cadherin expression was enhanced, and vimentin was reduced by the depletion of MMP2-AS1 (Figure 2(e)).

### 3.3. MMP2-AS1 Contributes to Proliferation, Migration, and Invasion of Renal Cell Carcinoma Cells by Inducing MMP2 Expression

**Figure 3:**
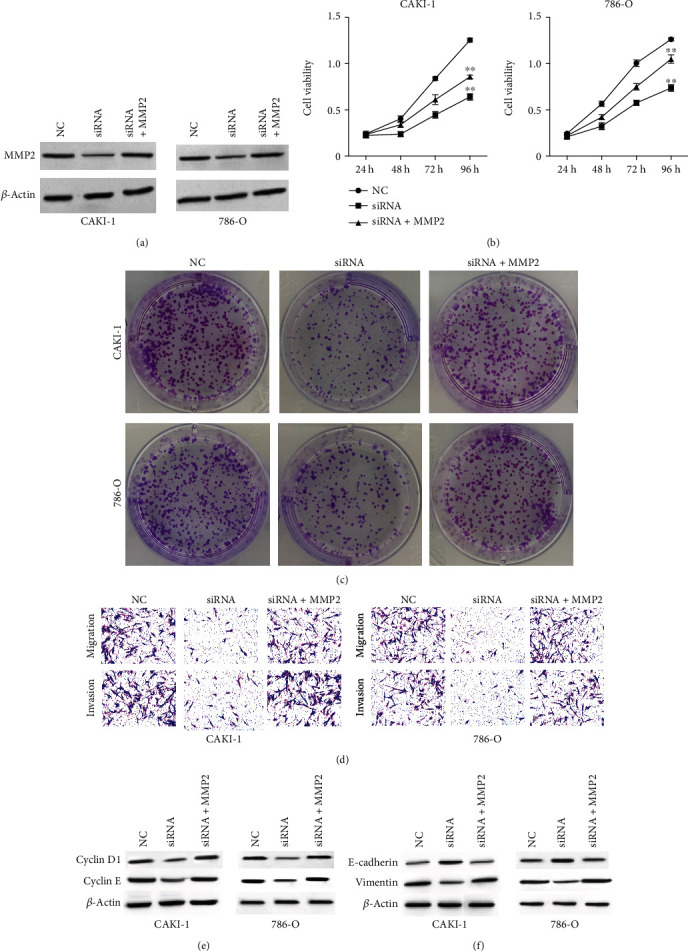
MMP2-AS1 contributes to proliferation, migration, and invasion of renal cell carcinoma cells by inducing MMP2 expression. (a–f) The CAKI-1 and 786-O cells were treated with MMP2-AS1 siRNA, or cotreated with MMP2-AS1 siRNA and MMP2 overexpressing plasmid. (a) The expression of MMP2 was examined by Western blot analysis. The cell viability was analyzed by CCK-8 assay. (c) The cell proliferation was detected by colony formation assay. (d) The cell migration and invasion were tested by transwell assay. (e, f) The expression of cyclin D1, cyclin E, E-cadherin, and vimentin was detected by Western blot. ^∗∗^*P* < 0.01.

Next, we observed that the expression of MMP2 was downregulated by the silencing of MMP2-AS1 in CAKI-1 and 786-O cells, in which the overexpression of MMP2 rescued the expression (Figure 3(a)). Significantly, the viability of CAKI-1 and 786-O cells was suppressed by the depletion of MMP2-AS1, which was rescued by the overexpression of MMP2 (Figure 3(b)). Meanwhile, the MMP2-AS1 depletion-inhibited colony formation numbers of CAKI-1 and 786-O cells were restored by the overexpression of MMP2 (Figure 3(c)). The knockdown of MMP2-AS1 reduced the ability of CAKI-1 and 786-O cell migration and invasion, while MMP2 overexpression could reverse the effect in the cells (Figure 3(d)). The overexpression of MMP2 reversed the effect of MMP2-AS1 depletion on cyclin D1, cyclin E, E-cadherin, and vimentin in the cells (Figures 3(e) and 3(f)).

### 3.4. MMP2-AS1 Promotes Tumor Growth of Renal Cell Carcinoma Cells by Enhancing MMP2 Expression

**Figure 4:**
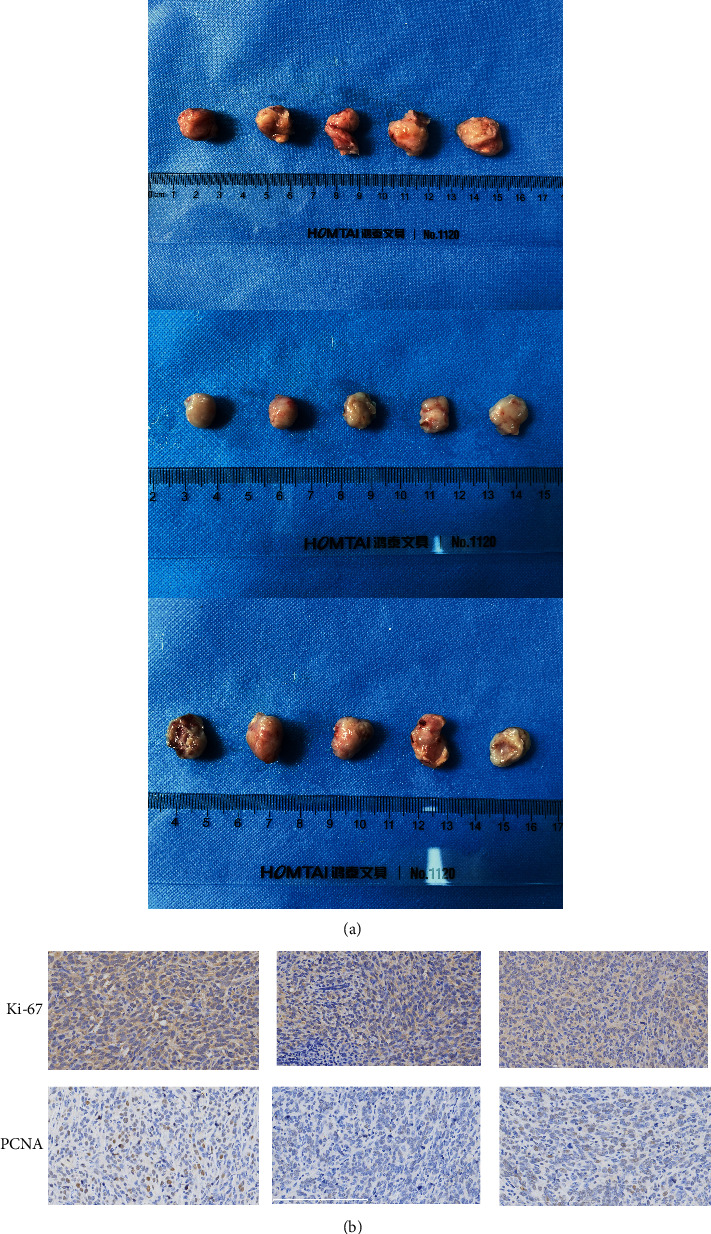
MMP2-AS1 promotes tumor growth of renal cell carcinoma cells by enhancing MMP2 expression. (a, b) The tumorigenesis analysis was performed in nude mice injected with CAKI-1 cells treated with MMP2-AS1 siRNA, or cotreated with MMP2-AS1 siRNA and MMP2 overexpressing plasmid. (a) The tumor images were shown. The levels of Ki-67 and PCNA were detected by IHC.

We then evaluated the correlation of MMP2-AS1 and MMP2 in the regulation of tumor growth of renal cell carcinoma cells *in vivo*. We observed that the tumor growth, along with the levels of Ki-67 and PCNA, were repressed by the depletion of MMP2-AS1, while the overexpression of MMP2 could reverse the effect in the nude mice (Figures 4(a) and 4(b)).

### 3.5. MMP2-AS1 Induces MMP2 Expression by Targeting miR-34c-5p

**Figure 5:**
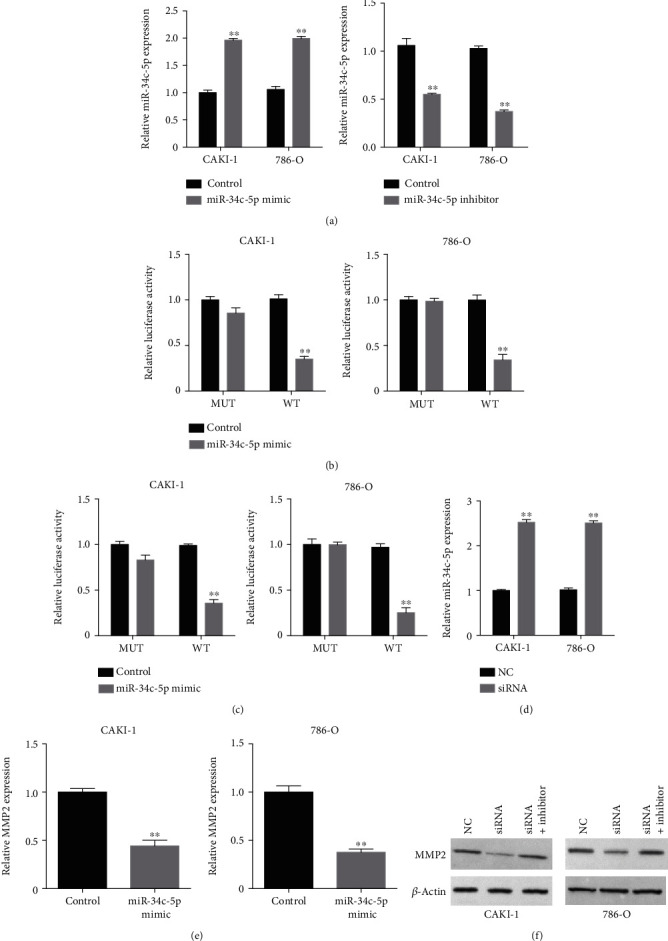
MMP2-AS1 induces MMP2 expression by targeting miR-34c-5p. (a) The CAKI-1 and 786-O cells were transfected with miR-34c-5p mimic or inhibitor. The expression of miR-34c-5p was measured by qPCR. (b, c) The CAKI-1 and 786-O cells were transfected with miR-34c-5p mimic. The luciferase activity of MMP2-AS1 and MMP2 3′UTR was measured by luciferase reporter gene assay. (d) The expression of miR-34c-5p was analyzed by qPCR in CAKI-1 and 786-O cells treated with MMP2-AS1 siRNA. (e) The expression of MMP2 was determined by qPCR in CAKI-1 and 786-O cells treated with miR-34c-5p mimic. (f) The expression of MMP2 was tested by Western blot analysis in CAKI-1 and 786-O cells treated with MMP2-AS1 siRNA, or co-treated with miR-34c-5p inhibitor. ^∗∗^*P* < 0.01.

Then, we explored the mechanism by which MMP2-AS1 enhanced MMP2 expression. We predicted the potential interaction of miR-34c-5p with both MMP2-AS1 and MMP2 and the overexpression of miR-34c-5p by mimic or the inhibition of miR-34c-5p by inhibitor were validated in CAKI-1 and 786-O cells (Figure 5(a)). The treatment of miR-34c-5p mimic reduced the luciferase activity of MMP2-AS1 and MMP2 3′UTR (Figures 5(b) and 5(c)). The depletion of MMP2-AS1 was able to enhance miR-34c-5p expression in CAKI-1 and 786-O cells (Figure 5(d)). The expression of MMP2 was inhibited by miR-34c-5p mimic in CAKI-1 and 786-O cells (Figure 5(e)). Importantly, the protein levels of MMP2 were downregulated by MMP2-AS1 knockdown, while the inhibitor of miR-34c-5p rescued the expression of MMP2 in CAKI-1 and 786-O cells (Figure 5(f)).

### 3.6. miR-34c-5p Suppresses Proliferation, Migration, and Invasion of Renal Cell Carcinoma Cells by Targeting MMP2 Expression

**Figure 6:**
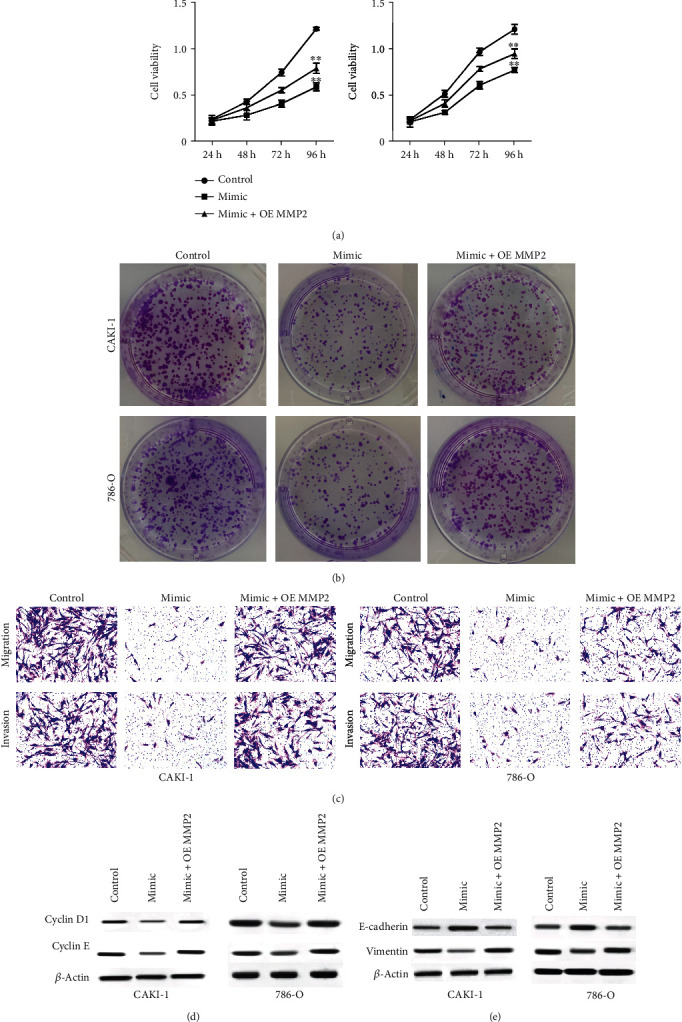
miR-34c-5p suppresses proliferation, migration, and invasion of renal cell carcinoma cells by targeting MMP2 expression. (a–c) The CAKI-1 and 786-O cells were treated with miR-34c-5p mimic, or cotreated with miR-34c-5p mimic and MMP2 overexpressing plasmid. (a) The cell viability was analyzed by CCK-8 assay. The cell proliferation was detected by colony formation assay. (c) The cell migration and invasion were tested by Transwell assay. (d, e) The expression of cyclin D1, cyclin E, E-cadherin, and vimentin was detected by Western blot. ^∗∗^*P* < 0.01.

Next, we observed that the viability of CAKI-1 and 786-O cells was inhibited by miR-34c-5p mimic, which was reversed by the overexpression of MMP2 (Figure 6(a)). Besides, miR-34c-5p-attenuated colony formation numbers of CAKI-1 and 786-O cells were rescued by the overexpression of MMP2 (Figure 6(b)). The treatment of miR-34c-5p mimic attenuated the ability of CAKI-1 and 786-O cell migration and invasion, while MMP2 overexpression could restore the ability in the cells (Figure 6(c)). The overexpression of MMP2 reversed the effect of miR-34c-5p mimic on cyclin D1, cyclin E, E-cadherin, and vimentin in the cells (Figures 6(d) and 6(e)).

### 3.7. MMP2-AS1 Contributes to Proliferation, Migration, and Invasion of Renal Cell Carcinoma Cells by Inducing MMP2 Expression

**Figure 7:**
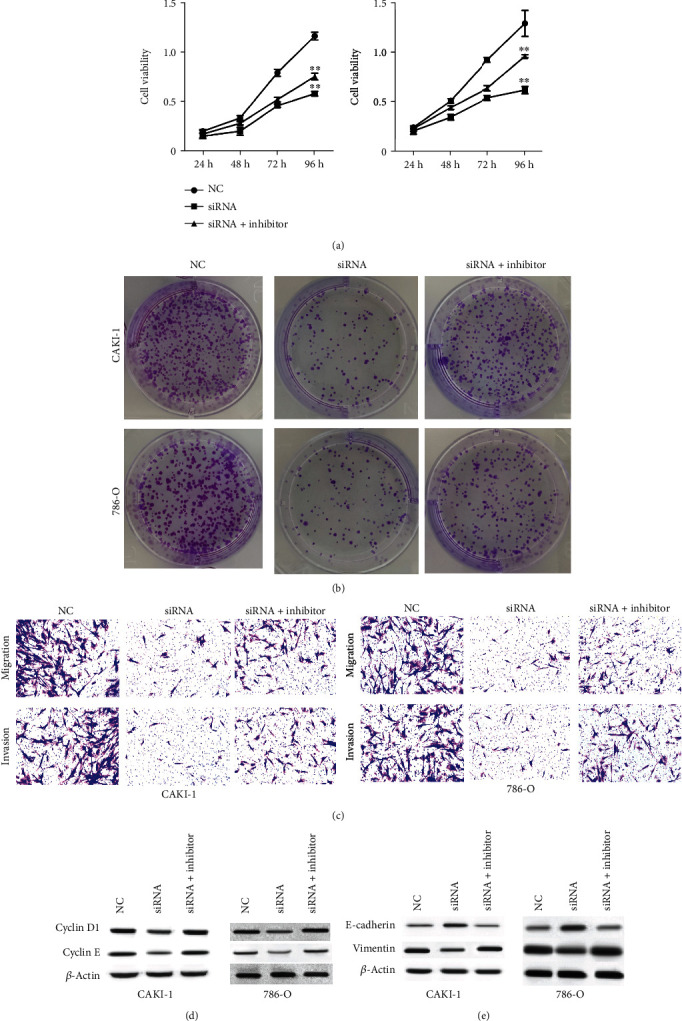
MMP2-AS1 contributes to proliferation, migration, and invasion of renal cell carcinoma cells by inducing MMP2 expression. (a–c) The CAKI-1 and 786-O cells were treated with MMP2-AS1 siRNA, or cotreated with miR-34c-5p inhibitor. (a) The cell viability was analyzed by CCK-8 assay. The cell proliferation was detected by colony formation assay. (c) The cell migration and invasion were tested by Transwell assay. (d, e) The expression of cyclin D1, cyclin E, E-cadherin, and vimentin was detected by Western blot. ^∗∗^*P* < 0.01.

Furthermore, we found that the viability of CAKI-1 and 786-O cells was inhibited by the depletion of MMP2-AS1, which was rescued by miR-34c-5p inhibitor (Figure 7(a)). In addition, the MMP2-AS1 depletion-attenuated colony formation numbers of CAKI-1 and 786-O cells were reversed by the inhibition of miR-34c-5p (Figure 7(b)). The silencing of MMP2-AS1 repressed the ability of CAKI-1 and 786-O cell migration and invasion, while miR-34c-5p inhibitor could reverse the effect in the cells (Figure 7(c)). The miR-34c-5p inhibitor reversed the effect of MMP2-AS1 depletion on cyclin D1, cyclin E, E-cadherin, and vimentin in the cells (Figures 7(d) and 7(e)).

## 4. Discussion

RCC is a prevalent malignancy of urinary system and shows low survival rate and high incidence of metastasis. lncRNAs have been identified as the crucial molecules in RCC development. Nevertheless, the function of lncRNA MMP2-AS1 in RCC remains obscure. In this study, we discovered the crucial effect of MMP2-AS1 on RCC progression and identified the potential mechanism.

The critical functions of lncRNAs have been reported in RCC progression in the previous studies. It has been reported that lncRNA miR4435-2HG promotes RCC malignant development by miR-513a-5p/KLF6 signaling [27]. lncRNA HOTAIRM1 is reduced in RCC and represses the hypoxia signaling [28]. lncRNA-SARCC inhibits RCC progression by regulating the androgen receptor/miRNA-143-3p axis [29]. Meanwhile, it has been found that miR-429/CRKL axis modulates RCC malignant development by SOS1/MEK/ERK/MMP2 axis [25]. G6PD contributes to RCC invasion by inducing MMP2 expression *via* ROS/MAPK signaling [26]. In the current work, we found that MP2-AS1 localized in both nucleus and cytoplasm of RCC cells using fluorescent in situ hybridization (FISH). Meanwhile, the cell viability and proliferation of RCC cells was remarkably reduced by the depletion of MMP2-AS1. The silencing of MMP2-AS1 remarkably attenuated the invasion and migration ability of RCC cells. Significantly, the expression of MMP2 was downregulated by the silencing of MMP2-AS1 in RCC cells, in which the overexpression of MMP2 rescued the expression. The MMP2-AS1 depletion-inhibited viability, proliferation, migration, and invasion of RCC cells were rescued by the overexpression of MMP2 *in vitro*. Consistently, the tumor growth of RCC cells were repressed by the depletion of MMP2-AS1 in the nude mice, while the overexpression of MMP2 could reversed this effect *in vivo*. Our data not only provide the evidence of the novel function of MMP2-AS1 in RCC progression but also elucidate the correlation of MMP2-AS1 with MMP2 in the development of RCC.

Previous studies have demonstrated the function of miR-34c-5p and MMP2 in cancer development. It has been reported that miRNA-34c-5p represses amphiregulin-stimulated ovarian cancer drug resistance and stemness by downregulating the AREG/EGFR/ERK axis [30]. lncRNA KCNQ1OT1 sponges miR-34c-5p to enhances osteosarcoma progression by ALDOA-enhanced aerobic glycolysis [31]. miR-34c-5p represses Notch1 and inhibits invasion and metastasis of cervical cancer [21]. miR-34c-5p regulates paclitaxel-induced apoptosis of lung cancer cells [32]. miR-34c-5p is correlated with the recurrence of laryngeal squamous cell carcinoma [33]. Moreover, it has been reported that SPAG5 contributes to metastasis of osteosarcoma by activating FOXM1/MMP2 signaling [34]. SPOCK2 regulates endometrial cancer cells by targeting MMP2 [35]. Exosome-transmitted circ_MMP2 enhances metastasis of hepatocellular carcinoma through inducing MMP2 [36]. Our mechanism investigations showed the potential interaction of miR-34c-5p with both MMP2-AS1 and MMP2. The treatment of miR-34c-5p mimic reduced the luciferase activity of MMP2-AS1 and MMP2 3′UTR. The depletion of MMP2-AS1 was able to enhance miR-34c-5p expression, and the expression of MMP2 was inhibited by miR-34c-5p mimic in RCC cells. The protein levels of MMP2 were downregulated by MMP2-AS1 knockdown, while the inhibitor of miR-34c-5p rescued the expression of MMP2 in the cells. The treatment of miR-34c-5p mimic attenuated the cell viability, proliferation, invasion, and migration of RCC cells, in which MMP2 overexpression could restore the phenotypes. Besides, MMP2-AS1 depletion-attenuated viability, proliferation, migration, and invasion of RCC cells were reversed by the miR-34c-5p inhibitor. In this study, we novelty explored the function of MMP2-AS1 in promoting RCC progression and identified the downstream miR-34c-5p/MMP2 axis. Our finding provides new insight into the mechanism by which MMP2-AS1 promotes RCC progression by targeting miR-34c-5p/MMP2 axis. Moreover, there are still some limitations in the current studies. For example, the miR-34c-5p/MMP2 axis may be just one of the mechanisms of MMP2-AS1 regulating RCC and other potential downstream factors should be explored in the future. Meanwhile, the clinical expression and association of MMP2, miR-34c-5p, and MMP2-AS1 should be evaluated in future studies.

We concluded that MMP2-AS1 contributed to progression of renal cell carcinoma by modulating the miR-34c-5p/MMP2 axis. MMP2-AS1 may serve as a potential therapeutic target in the treatment of RCC.

## Data Availability

The data used to support the findings of this study are included within the article.

## References

[1] Cohen H. T., McGovern F. J. (2005). Renal-cell carcinoma. *The New England Journal of Medicine*.

[2] Escudier B., Porta C., Schmidinger M. (2016). Renal cell carcinoma: ESMO clinical practice guidelines for diagnosis, treatment and follow-up^†^. *Annals of Oncology*.

[3] Vuong L., Kotecha R. R., Voss M. H., Hakimi A. A. (2019). Tumor microenvironment dynamics in clear-cell renal cell carcinoma. *Cancer Discovery*.

[4] Sonpavde G., Choueiri T. K., Escudier B. (2012). Sequencing of agents for metastatic renal cell carcinoma: can we customize therapy?. *European Urology*.

[5] Guttman M., Amit I., Garber M. (2009). Chromatin signature reveals over a thousand highly conserved large non-coding RNAs in mammals. *Nature*.

[6] Nagano T., Fraser P. (2011). No-nonsense functions for long noncoding RNAs. *Cell*.

[7] Liyanage K. I. P., Ganegoda G. U. (2017). Therapeutic approaches and role of ncRNAs in cardiovascular disorders and insulin resistance. *BioMed Research International*.

[8] Chen S., Ye H., Gong F. (2021). Ginsenoside compound K exerts antitumour effects in renal cell carcinoma via regulation of ROS and lncRNA THOR. *Oncology Reports*.

[9] Li L., Chang H. Y. (2014). Physiological roles of long noncoding RNAs: insight from knockout mice. *Trends in Cell Biology*.

[10] Zhang Y., Cao X. (2016). Long noncoding RNAs in innate immunity. *Cellular & Molecular Immunology*.

[11] Martens-Uzunova E. S., Bottcher R., Croce C. M., Jenster G., Visakorpi T., Calin G. A. (2014). Long noncoding RNA in prostate, bladder, and kidney cancer. *European Urology*.

[12] Schmitt A. M., Chang H. Y. (2016). Long noncoding RNAs in cancer pathways. *Cancer Cell*.

[13] Loewer S., Cabili M. N., Guttman M. (2010). Large intergenic non-coding RNA-RoR modulates reprogramming of human induced pluripotent stem cells. *Nature Genetics*.

[14] Guttman M., Donaghey J., Carey B. W. (2011). lincRNAs act in the circuitry controlling pluripotency and differentiation. *Nature*.

[15] Zhai W., Zhu R., Ma J. (2019). A positive feed-forward loop between LncRNA-URRCC and EGFL7/P-AKT/FOXO3 signaling promotes proliferation and metastasis of clear cell renal cell carcinoma. *Molecular Cancer*.

[16] Liu Y., Li X., Zhang C., Zhang H., Huang Y. (2020). LINC00973 is involved in cancer immune suppression through positive regulation of Siglec-15 in clear-cell renal cell carcinoma. *Cancer Science*.

[17] Bartel D. P. (2004). MicroRNAs: genomics, biogenesis, mechanism, and function. *Cell*.

[18] Bach D. H., Hong J. Y., Park H. J., Lee S. K. (2017). The role of exosomes and miRNAs in drug-resistance of cancer cells. *International Journal of Cancer*.

[19] Bayraktar R., Van Roosbroeck K. (2018). miR-155 in cancer drug resistance and as target for miRNA-based therapeutics. *Cancer and Metastasis Reviews*.

[20] Gao Z. Q., Wang J. F., Chen D. H. (2018). Long non-coding RNA GAS5 antagonizes the chemoresistance of pancreatic cancer cells through down-regulation of miR-181c-5p. *Biomedicine & Pharmacotherapy*.

[21] Wei H., Wang X., Niu X., Jiao R., Li X., Wang S. (2021). miR34c5p targets Notch1 and suppresses the metastasis and invasion of cervical cancer. *Molecular Medicine Reports*.

[22] Li N., Mao D., Cao Y., Li H., Ren F., Li K. (2018). Downregulation of SIRT6 by miR-34c-5p is associated with poor prognosis and promotes colon cancer proliferation through inhibiting apoptosis via the JAK2/STAT3 signaling pathway. *International Journal of Oncology*.

[23] Peng D., Wang H., Li L. (2018). miR-34c-5p promotes eradication of acute myeloid leukemia stem cells by inducing senescence through selective RAB27B targeting to inhibit exosome shedding. *Leukemia*.

[24] Kinget L., Roussel E., Verbiest A. (2021). MicroRNAs targeting HIF-2alpha, VEGFR1 and/or VEGFR2 as potential predictive biomarkers for VEGFR tyrosine kinase and HIF-2alpha inhibitors in metastatic clear-cell renal cell carcinoma. *Cancers (Basel)*.

[25] Wang J., Wang C., Li Q. (2020). miR-429-CRKL axis regulates clear cell renal cell carcinoma malignant progression through SOS1/MEK/ERK/MMP2/MMP9 pathway. *Biomedicine & Pharmacotherapy*.

[26] Zhang Q., Han Q., Yang Z. (2020). G6PD facilitates clear cell renal cell carcinoma invasion by enhancing MMP2 expression through ROS‑MAPK axis pathway. *International Journal of Oncology*.

[27] Zhu K., Miao C., Tian Y. (2020). lncRNA MIR4435-2HG promoted clear cell renal cell carcinoma malignant progression via miR-513a-5p/KLF6 axis. *Journal of Cellular and Molecular Medicine*.

[28] Hamilton M. J., Young M., Jang K. (2020). *HOTAIRM1* lncRNA is downregulated in clear cell renal cell carcinoma and inhibits the hypoxia pathway. *Cancer Letters*.

[29] Zhai W., Sun Y., Guo C. (2017). LncRNA-SARCC suppresses renal cell carcinoma (RCC) progression via altering the androgen receptor(AR)/miRNA-143-3p signals. *Cell Death and Differentiation*.

[30] Tung S. L., Huang W. C., Hsu F. C. (2017). miRNA-34c-5p inhibits amphiregulin-induced ovarian cancer stemness and drug resistance via downregulation of the AREG-EGFR-ERK pathway. *Oncogenesis*.

[31] Shen Y., Xu J., Pan X. (2020). LncRNA KCNQ1OT1 sponges miR-34c-5p to promote osteosarcoma growth via ALDOA enhanced aerobic glycolysis. *Cell Death & Disease*.

[32] Catuogno S., Cerchia L., Romano G., Pognonec P., Condorelli G., de Franciscis V. (2013). miR-34c may protect lung cancer cells from paclitaxel-induced apoptosis. *Oncogene*.

[33] Re M., Ceka A., Rubini C. (2015). MicroRNA-34c-5p is related to recurrence in laryngeal squamous cell carcinoma. *The Laryngoscope*.

[34] Li Z., Li H., Chen J. (2020). SPAG5 promotes osteosarcoma metastasis via activation of FOXM1/MMP2 axis. *The International Journal of Biochemistry & Cell Biology*.

[35] Ren F., Wang D., Wang Y., Chen P., Guo C. (2020). SPOCK2 affects the biological behavior of endometrial cancer cells by regulation of MT1-MMP and MMP2. *Reproductive Sciences*.

[36] Liu D., Kang H., Gao M. (2020). Exosome-transmitted circ_MMP2 promotes hepatocellular carcinoma metastasis by upregulating MMP2. *Molecular Oncology*.

